# Diagnostic Performance of Universal Transport Medium for Viral Polymerase Chain Reaction in Aqueous Humor Samples of Suspected Viral Uveitis: A Pilot Methods Study

**DOI:** 10.3390/ijms262010091

**Published:** 2025-10-16

**Authors:** Chungwoon Kim, Yoo-Ri Chung, Ji Hun Song, Young Joon Choi, Hae Rang Kim

**Affiliations:** Department of Ophthalmology, Ajou University School of Medicine, Suwon 16499, Republic of Korea; uni427@naver.com (C.K.); singtomorrow@gmail.com (Y.J.C.); khr1412@hanmail.net (H.R.K.)

**Keywords:** polymerase chain reaction, universal transport medium, uveitis, virus

## Abstract

We investigated the diagnostic efficacy of the universal transport medium™ (UTM^®^) as a transport medium for aqueous humor polymerase chain reaction (PCR) testing in patients with clinically suspected viral uveitis. This retrospective study included 31 patients (31 eyes) with presumed viral uveitis who underwent anterior chamber sampling and compared the viral detection rates between using UTM^®^ and conventional test tubes only. The positivity rate for any virus, including cytomegalovirus, herpes simplex virus, and varicella zoster virus, was significantly higher in the UTM^T®^ group than in the test tube group (64.3% vs. 23.5%, *p* = 0.033). Logistic regression analysis also revealed that the use of UTM^®^ significantly increased the PCR positivity rate (odds ratio, 5.850; 95% confidence interval, 1.222–27.994; *p* = 0.027). Thus, the use of UTM^®^ was associated with improved detection of causative pathogens in patients with presumed viral uveitis.

## 1. Introduction

Infectious uveitis remains a diagnostic challenge owing to its varied clinical manifestations and overlapping features among different etiologies. The common pathogens implicated in infectious viral uveitis include cytomegalovirus (CMV), herpes simplex virus (HSV), and varicella-zoster virus (VZV) [1]. Among the infectious pathogens for uveitis, viral anterior uveitis often presents with non-specific findings, such as granulomatous or non-granulomatous keratic precipitates, mild to moderate inflammation, and elevated intraocular pressure, rendering it difficult to distinguish clinically from other causes of anterior uveitis or masquerade syndromes [1]. However, the fact that at least 13~57% receiving empirical treatment change their therapeutic regimen once evidence of a viral etiology is confirmed suggests that establishing evidence beyond a clinical diagnosis of viral uveitis is of significant importance [2].

Polymerase chain reaction (PCR) analysis of ocular samples has been introduced and recommended as a standard diagnostic tool for aiding the etiologic diagnosis of viral anterior uveitis by Standardization of Uveitis Nomenclature (SUN) classification criteria [3,4,5]. Positive PCR results for CMV, HSV, or VZV have been suggested by the SUN Working Group as exclusion criteria for several systemic disease-associated anterior uveitis, such as juvenile idiopathic arthritis or spondyloarthritis [6,7]. However, their usefulness in clinical practice remains controversial. Real-world studies have revealed varied positive rates, ranging from 11% to 57% [2,8]. As an empirical diagnosis is frequently made based on the clinical history and examination findings, despite low interobserver agreement for viral anterior uveitis among uveitis experts, aqueous humor sampling is not frequently performed in patients with presumed viral anterior uveitis [2].

Increasing the detection rate of viruses has emerged as an important priority during pandemics. The influenza A H1N1 pandemic in 2009 and coronavirus disease 2019 (COVID-19) have resulted in the rapid development of transport media focusing on the optimal detection of viruses by PCR [9,10]. Universal Transport Medium™ (UTM^®^), originally developed for upper respiratory specimen collection, offers valuable advantages over conventional media by stabilizing viral nucleic acids and enhancing the pathogen detection rate, even in low-copy specimens. However, its application in ocular fluid sampling has not been extensively validated. Therefore, this study aimed to evaluate the diagnostic efficacy of UTM^®^ as a transport medium for aqueous humor PCR testing in patients with clinically suspected viral uveitis.

## 2. Results

### 2.1. Demographics and Ocular Characteristics

**Figure 1:**
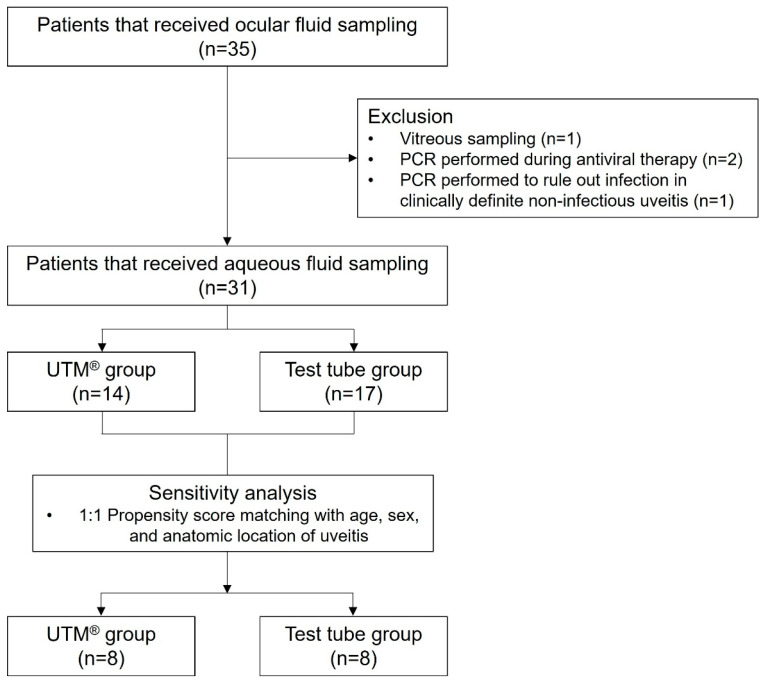
Flow diagram of patient inclusion and exclusion. PCR, polymerase chain reaction; UTM**^®^**, Universal Transport Medium™.

Among the 35 patients that were initially identified, 31 patients were finally included in this retrospective study (Figure 1).

A total of 31 patients were included in the analysis (see Supplementary File S1); their baseline demographic and diagnostic characteristics are summarized in Table 1. The mean age of patients was 52.9 ± 15.2 years; 17 patients (55%) were male. The mean intraocular pressure prior to sampling was 17.5 ± 8.8 mmHg. Twenty-one eyes (70%) showed keratic precipitates, whereas anterior chamber cells were present in 27 eyes (87%). Anterior uveitis was the most common pre-test diagnosis (*n* = 13), followed by retinitis or retinal vasculitis (*n* = 9), keratitis/endotheliitis (*n* = 6), and acute retinal necrosis (*n* = 3). PCR was performed for HSV (*n* = 21), VZV (*n* = 19), CMV (*n* = 26), Epstein–Barr virus (EBV, *n* = 4), and rubella virus (*n* = 2).

Analysis of clinician preference showed no significant difference in the frequency of UTM^®^ use between anterior-segment specialists (cornea/glaucoma) and posterior-segment specialists (retina/uveitis) (50% vs. 43%, *p* = 0.709 by chi-square test). However, we identified a clear temporal pattern in media selection: conventional test tubes were exclusively used before the COVID-19 pandemic (i.e., until 2019), while both methods were employed from 2020 onwards (11 vs. 14 cases).

### 2.2. Viral Detection

The aqueous humor samples were transported using either UTM^®^ (*n* = 14, 45%) or conventional sterile test tubes (*n* = 17, 55%). The UTM^®^ was more frequently used in uveitis cases mainly involving anterior segment (12/14, 85.7% of cases), whereas the conventional test tube was more used in posterior uveitis cases (10/17, 58.8% of cases). This different distribution of presumed diagnosis by transport medium was statistically significant (*p* = 0.024 by Fisher’s exact test).

**Figure 2:**
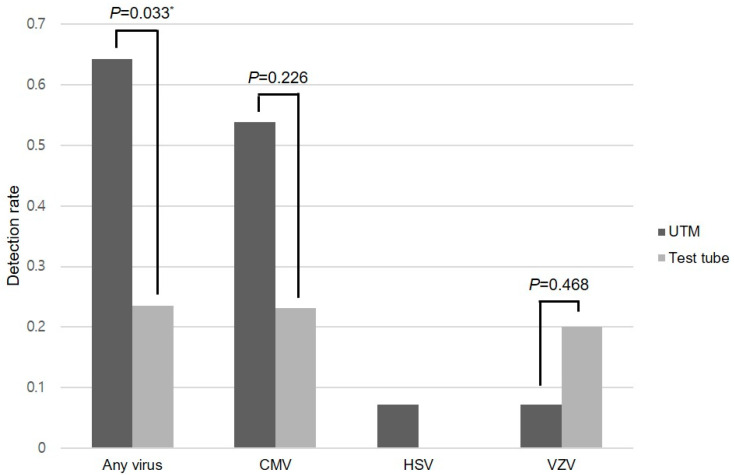
Comparison of the viral detection rates for CMV, HSV, and VZV between the Universal Transport Medium™ (UTM**^®^**) and conventional test tube. The PCR positivity rate for “any virus” was significantly higher in the UTM^®^ group than the test tube group using Fisher’s exact test, while no significant difference was observed for CMV. Individual-virus comparisons were not significant for VZV due to small numbers and not estimable for HSV due to zero events in the test tube group. The bar graph displays the proportion of positive results using aqueous humor samples. * *p* < 0.05 by Fisher’s exact test (applied to “any virus” comparison). CMV, cytomegalovirus; HSV, herpes simplex virus; VZV, varicella-zoster virus.

EBV and rubella virus were not detected in the tested specimens. The PCR positivity rate for the other three viruses (CMV, HSV, and VZV) was significantly higher in the UTM^®^ group than in the test tube group (64.3% vs. 23.5%, *p* = 0.033), as shown in Figure 2. The UTM^®^ group had a 40.8% higher absolute risk of detection compared to the test tube group (95% confidence interval [CI] 8.6–73.0%). This corresponds to an odds ratio of 5.850 (95% CI 1.222–27.994, *p* = 0.027) favoring UTM^®^.

The subgroup analysis stratified by individual viruses demonstrated higher positivity rates for HSV and CMV in the UTM^®^ group than in the test tube group. The CMV positivity was notably higher in the UTM^®^ group (53.8% vs. 23.1%); however, we were unable to detect a statistically significant difference due to the limited number of cases available in the subgroup analyses (*p* = 0.226). Statistical comparison of HSV was not feasible owing to the absence of positive cases in the test tube group. Regarding VZV, statistically significant differences were not evident owing to the limited number of positive samples in each group (Figure 2 and Table 2).

Data on quantitative viral load was available for CMV-positive cases, whereas the results were provided only qualitatively (positive or negative) for HSV and VZV. Individual viral loads for each CMV-positive patient can be found in Supplementary File S1. Among CMV-positive cases, the distribution was highly right-skewed with an overall mean viral load of 962,572 IU/mL (range: 258–9,140,000 IU/mL). The mean viral load for CMV was 20,548 IU/mL in the UTM^®^ group and 3,160,630 IU/mL in the test tube group; however, no statistically significant difference was detected due to the limited number of cases and skewed data (*p* = 0.210, Mann–Whitney U test) (Table 3). The Hodges–Lehmann estimate of the median difference was −337,910 IU/mL (95% CI −9,139,625–46,310).

Two patients with anterior uveitis underwent repeated aqueous humor sampling. CMV was detected in the second paracentesis with UTM^®^ in one patient with hypertensive anterior uveitis, whereas the other patient revealed no viral detection despite one sampling with a conventional test tube and two samples with UTM^®^.

Logistic regression analysis revealed that UTM^®^ played a significant role in viral detection (odds ratio [OR] 5.850, 95% confidence interval [CI], 1.222–27.994; *p* = 0.027). The anatomical classification of uveitis whether involving mainly anterior (keratitis, endotheliitis, or anterior uveitis) or posterior segment (retinitis, retinal vasculitis, or acute retinal necrosis) was not a significant factor (OR 1.018, 95% CI 0.235–4.407, *p* = 0.981), neither were age or sex (Table 4). The use of UTM^®^ remained a significant factor when adjusted for age and sex (Model 1, OR 7.211, 95% CI 1.174–44.294, *p* = 0.033) and also when adjusted for age, sex, and anatomic location of uveitis (Model 2, OR 13.669, 95% CI 1.106–168.896, *p* = 0.041). However, it was no longer significant when fully adjusted for ocular confounders and the aforementioned factors (Model 3, OR 33.108, 95% CI 0.765–1432.485, *p* = 0.069), possibly due to reduced statistical power from including multiple covariates in a small sample. No procedure-related complications, such as hyphema, infection, or intraocular pressure-related events, were reported during or after the aqueous sampling procedures.

### 2.3. Sensitivity Analyses

In the subgroup analysis restricted to anterior-segment inflammation cases (*n* = 19), the overall viral positivity rate remained higher in the UTM^®^ group compared to the test tube group, though this difference did not reach statistical significance (58.3% vs. 14.3%, *p* = 0.147). Propensity scores were estimated based on age, sex, and anatomic location of uveitis, and 1:1 nearest-neighbor matching was performed with a caliper of 0.2. The matched analysis, which included 8 cases in each group, demonstrated a consistent trend toward higher viral detection in the UTM^®^ group, although the difference did not reach statistical significance (62.5% vs. 25.0%, *p* = 0.315).

## 3. Discussion

In this study, we demonstrated that the use of UTM^®^ as a transport medium significantly improved viral detection rates in aqueous humor PCR testing, with an overall positivity rate of 64.3% compared to 23.5% with conventional test tubes (*p* = 0.033). This nearly three-fold increase in detection rate has important clinical implications, particularly given that aqueous humor PCR positivity often leads to treatment modifications in 13–57% of uveitis patients [2,10,11]. Despite this clinical relevance, the utilization of aqueous humor sampling remains low among uveitis specialists, likely due to the historically limited PCR positivity rates of only 28–42% for viral pathogens [11,12,13]. Our findings suggest that optimizing pre-analytical factors, specifically the use of appropriate transport media, may address these diagnostic limitations.

The conventional transfer of ocular samples via plain sterile tubes may not optimally preserve nucleic acids, particularly in small-volume samples. Degradation of viral deoxyribonucleic acid (DNA) or ribonucleic acid (RNA) during transport or processing can lead to false negative results and missed diagnoses. This study focused on aqueous humor sampling and transport and demonstrated that the use of UTM^®^ improved the viral detection rate from aqueous humor PCR testing in patients with clinically suspected viral uveitis. Our results demonstrated that the overall PCR positivity rate for herpes viridae viruses, including CMV, HSV, and VZV, was significantly higher when UTM^®^ was used as the transport medium than when conventional sterile test tubes without medium were used. UTM^®^ is optimized for the preservation of viruses, chlamydia, and mycoplasmas, and contains nucleic acid stabilizers and antimicrobial agents that protect viral DNA from degradation, especially during delays between sampling and analysis [14]. Originally developed for respiratory virus detection, UTM^®^ has been widely validated during the COVID-19 pandemic to maintain viral RNA integrity under various storage and transport conditions [9,14]. Our transfer method of mixing the aqueous humor specimen with 2 mL of UTM^®^ may lead to significant dilution and could be a potential confounder. However, although the PCR assay used in this study was not validated directly for this dilution method and all detection thresholds were applied identically to both groups, previous studies on the detection of SARS-CoV-2 and other viruses revealed that viral detection remained comparably effective across various specimens and transport conditions using UTM^®^ [8,15]. While the impact of dilution of ocular specimens with UTM^®^ on PCR positivity could not be evaluated in our retrospective study, the aforementioned studies suggest that improved overall detection is plausible despite dilution. When applied to ocular specimens, its stabilizing properties may increase the PCR sensitivity, particularly in samples with a low viral copy number. Therefore, improved detection using UTM^®^ sampling may offer significant clinical benefits. Early and accurate identification of viral pathogens enables more targeted therapy, prevents unnecessary immunosuppression, and may reduce the risk of irreversible vision loss in rapidly progressive infections, such as acute retinal necrosis or CMV retinitis. In patients with overlapping features or atypical presentations, even a modest increase in PCR sensitivity can contribute to timely therapeutic decision-making and improve outcomes. Taken together, the use of UTM^®^ as a transport medium may ameliorate some of the limitations inherent to conventional aqueous humor PCR testing.

CMV is the predominant cause of anterior uveitis in immunocompetent individuals, particularly in Asia [16]. Local reactivation of latent CMV presumably causes anterior uveitis, presenting as Posner–Schlossman syndrome in the acute form and Fuchs iridocyclitis in the chronic form [16]. The clinical features of CMV-related anterior uveitis include relatively mild anterior chamber inflammation but significantly elevated intraocular pressure [12,16]. The relatively high CMV detection rate in this study may be related to the high prevalence of CMV-associated uveitis in Asia. Although not statistically significant, the use of UTM^®^ resulted in a higher detection rate of CMV than the use of conventional test tubes. Meanwhile, HSV and VZV positivity rates were generally low; therefore, there was no difference in the use of UTM^®^. Importantly, while our data show varying detection rates for CMV, HSV, and VZV, these differences should not be interpreted as evidence of virus-specific performance of UTM^®^ or clinical superiority for detecting particular viral pathogens. Rather, these variations likely reflect the limited sample size and the inherent challenges of subgroup analyses in a pilot study. These findings might also be influenced by the differences in the natural course and latency of each virus, and variability in the viral load in the anterior chamber at the time of sampling. Therefore, further studies involving larger numbers of patients are warranted.

Despite these promising findings, this study has certain limitations. The retrospective design and single-center setting may have limited generalizability and account for potential selection bias. The relatively small sample size (*n* = 31), particularly within virus-specific subgroups, might have underpowered some comparisons. Therefore, the results of our study require further validation in a large, prospective study to confirm its generalizability. Another limitation is that the utilization rate of UTM^®^ varied depending on the diagnosis, which may lead to a potential data bias. The variation in the distribution of anatomical classifications of uveitis between the UTM^®^ group and the test tube group was also regarded as a limitation attributable to the small sample size. Further investigation of serial dilution with UTM^®^ as well as comparison with a control buffer (e.g., saline) would provide additional information on the impact of dilution on PCR sensitivity that was not evaluated in this retrospective study. We also could not assess the exact time from sampling to the PCR analysis, although PCR testing was performed within 24 h of sample collection in all cases according to the testing laboratory protocols. Detailed information regarding specific per-sample time intervals (minutes from tap to refrigeration, exact duration at 4 °C, and individual transport times) was not available in the medical records due to the retrospective nature of the study. Additional consideration should be given to the fact that this study could not include data on the quantitative viral loads or cycle threshold (Ct) values in viruses other than CMV, which could provide additional insights into the effects of transport media and conditions on assay sensitivity. As several assays were performed at external laboratories, some analytical parameters (e.g., LOQ, linear range, internal control design) remained unavailable despite detailed methodological specifications for all PCR assays in Appendix A, which may limit the depth of inter-virus comparisons. Finally, while sample input volumes were standardized within each virus-specific assay, inter-laboratory differences in PCR platforms may have resulted in varying input requirements across viruses, limiting direct inter-virus comparisons.

## 4. Materials and Methods

### 4.1. Patient Enrollment and Study Design

In this retrospective, observational, single-center study, the medical records of patients who underwent aqueous humor sampling for viral PCR between March 2014 and January 2025 at Ajou University Hospital (Suwon, Republic of Korea) were reviewed. This study complied with the Declaration of Helsinki and was approved by the Institutional Review Board (IRB) of Ajou University Hospital (approval no. AJOUIRB-DB-2025-396). The need for informed consent was waived owing to the retrospective nature of the study. Demographic data, clinical characteristics, diagnostic categories, and PCR test results were collected from medical records. The STROBE checklist is provided in Supplementary File S2.

The initial diagnosis was based on clinical findings and disease progression. All patients were diagnosed and managed by the uveitis specialists in our department following standardized clinical criteria. The primary outcome was the overall viral detection (any positive PCR result for CMV, HSV, or VZV) stratified by transport medium. The decision to use UTM^®^ versus conventional test tube was based on the gradual adoption of UTM^®^ in our practice over the study period rather than clinician preference. Aqueous humor PCR testing was subsequently conducted according to the clinical judgment of the attending physician (stratified as anterior segment surgeon vs. posterior segment surgeon). Ocular diagnoses were classified into the following categories: (1) corneal involvement, such as keratitis or endotheliitis; (2) anterior uveitis; (3) retinitis or posterior uveitis; (4) ARN. The exclusion criteria were as follows: (1) PCR performed using vitreous samples; (2) PCR performed while antiviral medications in use; (3) PCR performed to exclude patients with highly suspected non-infectious uveitis. When multiple sampling was performed for diagnosis, it was considered a separate case unless the exclusion criteria were fulfilled.

### 4.2. Procedure

**Figure 3:**
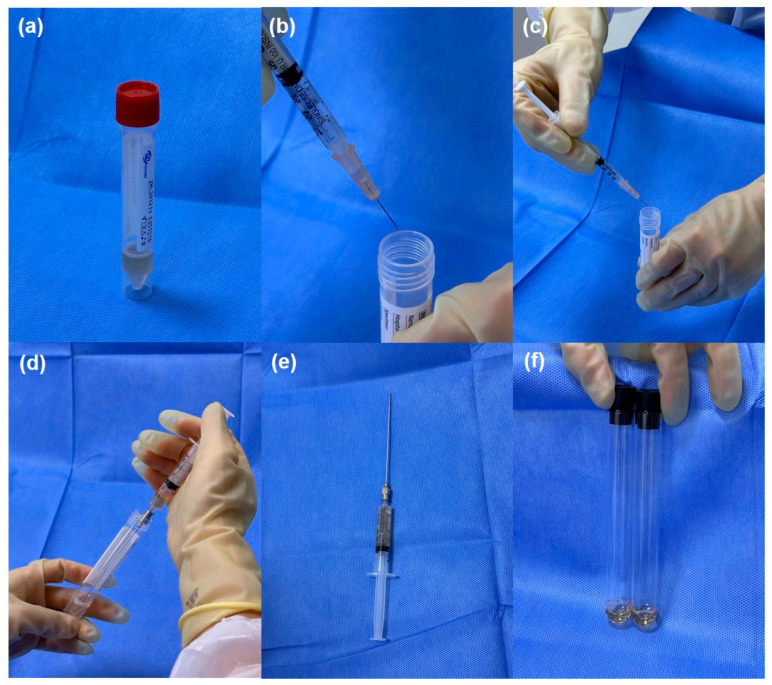
Step-by-step procedure for aqueous humor sampling using the Universal Transport Medium (UTM^®^). (**a**) UTM^®^ (Cogan Universal Transport Medium, Copan Diagnostics, CA) vial. (**b**,**c**) Mixture of aqueous humor collected via anterior chamber paracentesis under topical anesthesia and sterile conditions to UTM^®^ vial. (**d**) The entire mixture of aqueous fluid and UTM^®^ was aspirated using a 22-gauge spinal needle. (**e**) The final sample, now diluted to approximately 2 mL, demonstrates increased volume post-mixing. (**f**) The final sample was aliquoted into standard test tubes for polymerase chain reaction testing.

The groups were classified by the transport medium. Aqueous fluid sampling was performed with topical anesthesia under sterile conditions, using a 30-gauge needle to extract 0.05–0.2 mL of aqueous humor. Patients in the test tube group had aqueous humor samples transferred directly into an empty sterile test tube without any transport medium. For those classified as UTM^®^ group, the collected aqueous humor was mixed thoroughly with 2 mL of UTM^®^ (Cogan Universal Transport Medium, Copan Diagnostics, Murrieta, CA, USA), immediately after sampling. This dilution was achieved by directly injecting an aqueous sample into a UTM^®^ vial using a sterile syringe. The resulting mixture was gently agitated to ensure homogeneity. The collection flow of the UTM^®^ is shown in Figure 3. Samples were immediately transported to the laboratory for viral PCR analysis. In some cases, transportation or processing delay was unavoidable owing to the institutional workflow, during which time the sample was stored temporarily at 4 °C until PCR analysis. This was applied to both groups.

PCR testing was performed at standardized laboratories for each virus (CMV at Ajou University Hospital, Suwon, Republic of Korea; HSV/VZV at Green Cross Laboratories, Yongin, Republic of Korea; EBV/rubella at Seoul Clinical Laboratories, Seoul, Republic of Korea), with identical protocols applied to both UTM^®^ and conventional test tube groups for each specific pathogen. Real-time PCR was performed within 24 h of sample collection according to the protocols recommended by the testing laboratory and reagent manufacturers. The samples were analyzed for HSV, VZV, CMV, EBV, and rubella virus DNA based on clinical suspicion. Assay-specific detection thresholds/cut-offs by virus were as follows: (1) for CMV, cobas^®^ CMV (Roche Diagnostics International AG, Rotkreuz, Switzerland), limit of detection 20.6 IU/mL (95% CI, 17.9–24.3); (2) for HSV, BioCore HSV 1/2 Real-Time PCR Kit (Biocore Co., Ltd., Seoul, Republic of Korea), limit of detection 155 copies/mL for HSV1 and 163 copies/mL for HSV2; (3) for VZV, BioCore VZV Real-Time PCR Kit (Biocore Co., Ltd., Seoul, Republic of Korea), limit of detection 197 copies/mL; (4) for EBV, Alinity m EBV AMP Kit (Abbott, Abbott Park, IL, USA), limit of detection 50 IU/mL; (5) for rubella, BioCore Rubella Real-Time PCR Kit (Biocore Co., Ltd., Seoul, Republic of Korea), qualitative assay with a manufacturer-validated positivity cut-off of Ct = 45 (IU/mL calibration not available). The thresholds were applied identically to both groups, regardless of the transport method used (UTM^®^ or conventional test tube). PCR results were reported quantitatively (by IU/mL) for CMV and qualitatively (positive or negative) for HSV, VZV, EBV, and rubella, which were used to support the clinical diagnosis of viral uveitis. Further technical details of the PCR assays, including extraction methods, target genes, detection thresholds, and internal controls, are provided in Appendix A.

### 4.3. Statistical Analysis

Data was analyzed using the SPSS software (version 22.0; IBM Corp., Armonk, NY, USA). Categorical variables, including PCR positivity rates, were compared between the groups using chi-square test or Fisher’s exact test. Quantitative data between the groups were compared using Mann–Whitney U test. Logistic regression analysis was used to investigate significant factors for positive viral detection, which are presented as ORs with 95% CI. Potential confounders were adjusted in statistical models as follows: model 1, age and sex; model 2, age, sex, and anatomic location of main inflammation; model 3, fully adjusted with age, sex, anatomic location of main inflammation, intraocular pressure, keratic precipitates, and anterior chamber cells (see Supplementary File S3).

Sensitivity analysis was performed using a subgroup analysis restricted to anterior-segment presentations. Propensity scores were estimated based on age, sex, and the anatomic location of uveitis, and 1:1 matching of the UTM^®^ group and the test tube group was performed with a caliper of 0.2. *p*-values < 0.05 were considered statistically significant.

## 5. Conclusions

In this pilot study, the use of UTM^®^ as a transport medium was associated with improved overall detection rates of viral PCR in patients with suspected viral uveitis. While this represents a potentially useful approach for enhancing diagnostic sensitivity, prospective validation with larger sample sizes is required to confirm its impact on diagnostic utility and therapeutic outcomes before routine implementation in clinical practice. Future prospective validation studies should include larger sample sizes, co-primary outcomes, harmonized pre-analytics, and standardized PCR protocols across multiple centers. Incorporating quantitative PCR analysis and exploring other transport media or preservation strategies may further improve diagnostic reliability.

## Figures and Tables

**Table 1:** Demographic and ocular characteristics of the included patients.

Variable	*n* (%) or Mean ± SD
No. of patients	31
Age (years)	52.9 ± 15.2
Sex, female	17 (55%)
Ocular findings	
Intraocular pressure (mmHg)	17.5 ± 8.8
Keratic precipitates	21 (68%)
Anterior chamber cells	27 (87%)
Ocular diagnosis	
Keratitis/Endotheliitis	6 (19%)
Anterior uveitis	13 (42%)
Retinitis/Retinal vasculitis	9 (29%)
Acute retinal necrosis	3 (10%)
Use of UTM	14 (45%)
Use of conventional test tube	17 (55%)
PCR tests performed	
Cytomegalovirus	26 (84%)
Herpes simplex virus	21 (68%)
Varicella-zoster virus	19 (61%)
Epstein–Barr virus	4 (13%)
Rubella virus	2 (6%)

PCR, polymerase chain reaction; SD, standard deviation; UTM**^®^**, Universal Transport Medium™.

**Table 2:** Polymerase chain reaction positivity rates stratified by virus.

Virus	UTM^®^	Test Tube	*p*-Value ^†^
Cytomegalovirus	7/13 (53.8%)	3/13 (23.1%)	0.226
Herpes simplex virus	1/14 (7.1%)	0/7 (0%)	N/A ^‡^
Varicella-zoster virus	1/14 (7.1%)	1/5 (20.0%)	0.468 *

^†^ *p* value by Mann–Whitney U-test. ^‡^ Statistical comparison not meaningful because of zero events in the test tube group. * Cautious interpretation needed due to limited number of cases in test tube group. UTM**^®^**, Universal Transport Medium™.

**Table 3:** Distribution of cytomegalovirus viral load (IU/mL) by test group.

Test Group	*n*	Mean ± SD	Min–Max	Median (IQR)
All patients	10	962,572 ± 2,875,170	258–9,140,000	3735 (147,614)
UTM**^®^**	7	20,548 ± 33,456	258–85,500	2090 (47,825)
Test tube	3	3,160,630 ± 5,181,045	1890–9,140,000	340,000 (9,138,110)

IQR, interquartile range; SD, standard deviation; UTM^®^, Universal Transport Medium™. Data are presented as IU/mL.

**Table 4:** Logistic regression analysis for polymerase chain reaction positivity rates stratified by virus.

Variable	OR (95% CI)	*p*-Value
Age	1.069 (1.000–1.143)	0.051
Sex (female)	0.625 (0.147–2.664)	0.525
Use of UTM^®^	5.850 (1.222–27.994)	0.027 *
Anatomic location of uveitis (anterior segment)	1.018 (0.235–4.407)	0.981
Intraocular pressure	0.944 (0.858–1.040)	0.242
Presence of keratic precipitates	4.400 (0.749–25.842)	0.101
Presence of anterior chamber cells	2.400 (0.221–26.116)	0.472

CI, confidence interval; OR, odds ratio; UTM^®^, Universal Transport Medium™. * *p* < 0.05, logistic regression analysis.

## Data Availability

The comprehensive data used in this study are supplied as Supplementary Materials (Supplementary File S1).

## Supplementary Materials

The following supporting information can be downloaded at: https://www.mdpi.com/article/10.3390/ijms262010091/s1.

## Abbreviations

The following abbreviations are used in this manuscript:

CI	Confidence interval
CMV	Cytomegalovirus
COVID-19	Coronavirus disease 2019
Ct	Cycle threshold
DNA	Deoxyribonucleic acid
EBV	Epstein–Barr virus
HSV	Herpes simplex virus
OR	Odds ratio
PCR	Polymerase chain reaction
RNA	Ribonucleic acid
SUN	Standardization of Uveitis Nomenclature
UTM	Universal transport medium
VZV	Varicella-zoster virus

## Appendix A

### Appendix A.1. Molecular Assay Characteristics for CMV PCR

- Extraction kits and platforms: All specimens were processed on fully automated cobas^®^ 6800 Systems (Roche Diagnostics, Basel, Switzerland), which integrate nucleic acid extraction, purification, amplification, and detection. No external extraction kit was used.
- Target gene: Conserved region of the UL54 DNA polymerase gene of human cytomegalovirus (CMV).
- Positivity thresholds and quantification range: Results were reported in IU/mL, traceable to the WHO International Standard for CMV. Results were categorized as: not detected, <LLoQ, within quantifiable range, or >ULoQ. The lower limit of detection (LOD) was 20.6 IU/mL (95% CI, 17.9–24.3); the lower limit of quantification (LLoQ) was 34.5 IU/mL; and the upper limit of quantification (ULoQ) was 1.0 × 10^7^ IU/mL. The validated linear range extended from 2.45 × 10^1^ to 1.34 × 10^7^ IU/mL. According to the manufacturer, detection of CMV DNA above the LOD (20.6 IU/mL) is reported as positive, whereas results ≥ LLoQ (34.5 IU/mL) are considered within the validated quantifiable range.
- Internal controls: Each run included an exogenous DNA quantitation standard (DNA-QS) spiked into all samples prior to extraction, along with manufacturer-provided controls (CMV Low Positive Control, CMV High Positive Control, and Normal Human Plasma Negative Control).
- Sample input volumes: A minimum of 525 µL of specimen was required, of which 350 µL was aspirated into the cobas secondary tube for analysis. The same input requirements were applied to aqueous humor samples, and identical input volumes were maintained across all media and laboratories. Although the cobas CMV assay is formally validated for EDTA plasma, aqueous humor was processed under the same protocol; this off-label application was consistently implemented and is acknowledged in the main text as a study limitation.

### Appendix A.2. Molecular Assay Characteristics for Externally Performed PCR Assays (HSV, VZV, EBV, and Rubella Virus)

- Extraction kits and platforms: HSV and VZV assays were performed at Green Cross Laboratories (Yongin, Republic of Korea).
  Nucleic acids were extracted using the MagNA Pure 96 system (Roche Diagnostics) with the MagNA Pure 96 DNA and Viral NA Small Volume Kit.
  Subsequent amplification was performed on different PCR platforms: HSV testing was conducted using the CFX96 real-time PCR system with the HSV 1/2 Real-Time PCR Kit (Biocore Co., Seoul, Republic of Korea), while VZV testing was performed on the ProFlex conventional PCR platform using the VZV PCR Real-Time PCR Kit (Biocore Co., Ltd., Seoul, Republic of Korea).
  EBV and rubella virus assays were performed at Seoul Clinical Laboratories (Seoul, Republic of Korea).
  For EBV, extraction and amplification were conducted using the Alinity m Sample Preparation Kit (Abbott, Abbott Park, IL, USA) on the Alinity m platform, which integrates extraction, amplification, and detection.
  For rubella virus, extraction was carried out using the MagNA Pure 96 system with the MagNA Pure 96 DNA and Viral NA Small Volume Kit, followed by amplification on the SLAN 96P real-time PCR platform with the Rubella Real-Time PCR Kit (Biocore Co., Ltd., Seoul, Republic of Korea).
- Target gene: The BioCore HSV 1/2 Real-Time PCR Kit (Biocore Co., Seoul, Republic of Korea) amplifies the UL30 DNA polymerase gene conserved across HSV-1 and HSV-2.
  The BioCore VZV PCR Kit (Biocore Co., Ltd., Seoul, Republic of Korea) targets the ORF62 gene, a well-conserved immediate-early regulatory region of the VZV genome.
  The Alinity m EBV AMP Kit (Abbott, Abbott Park, IL, USA) amplifies the BamHI W region of the EBV genome.
  The BioCore Rubella Real-Time PCR Kit (Biocore Co., Ltd., Seoul, Republic of Korea) targets the E1 envelope glycoprotein gene of rubella virus.
- Positivity thresholds and quantification range: For HSV, the assay had a limit of detection (LOD) of 155 copies/mL for HSV-1 and 163 copies/mL for HSV-2. For VZV, the limit of detection was 197 copies/mL. For EBV, the limit of detection was 50 IU/mL, traceable to the WHO International Standard, and results ≥ 50 IU/mL were reported as positive. For rubella virus, this qualitative assay had a manufacturer-validated positivity cut-off of Ct ≤ 45. Additional assay performance characteristics such as the linear quantification range or LOQ were unavailable.
- Internal controls: Each assay included manufacturer-provided internal controls to monitor extraction and amplification efficiency. However, detailed specifications of these internal control materials were not disclosed by the laboratories.
- Sample input volumes: For HSV, VZV, and rubella virus assays, 200 µL of sample was used for extraction, while 800 µL was used for EBV testing. Identical input volumes were maintained across transport media (UTM^®^ vs. test tube) within each virus-specific assay. Although these assays were originally validated for use with plasma, serum, or cerebrospinal fluid specimens, the same protocols were applied to aqueous humor samples to ensure standardized processing across all media. This represents an off-label application, which was implemented consistently and is acknowledged in the main text as a study limitation.
