# Supplementary figures and images for: Diagnostic Performance of Universal Transport Medium for Viral Polymerase Chain Reaction in Aqueous Humor Samples of Suspected Viral Uveitis: A Pilot Methods Study
Source: Int J Mol Sci. 2025 Oct 16;26(20):10091. doi: 10.3390/ijms262010091 (PMC12564773; doi:10.3390/ijms262010091)

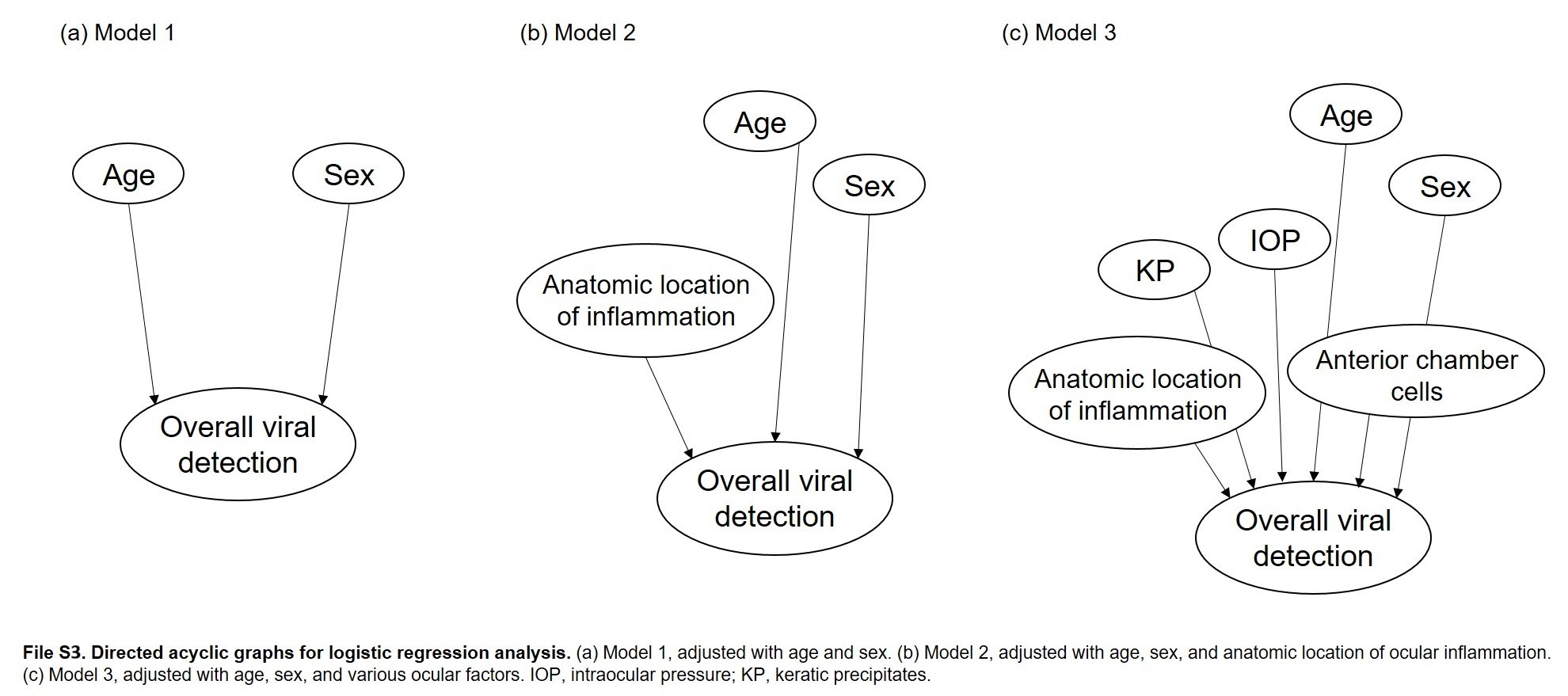

Supplement: Supplementary file 1 [file ijms-26-10091-s001.zip › File S3.jpg]
